# alpha-Actinin interacts with rapsyn in agrin-stimulated AChR clustering

**DOI:** 10.1186/1756-6606-1-18

**Published:** 2008-12-03

**Authors:** G Clement Dobbins, Shiwen Luo, Zhihua Yang, Wen C Xiong, Lin Mei

**Affiliations:** 1 Program of Developmental Neurobiology, Institute of Molecular Medicine and Genetics, and Department of Neurobiology, Medical College of Georgia, Augusta, Georgia 30912, USA; 2 Department of Neurobiology, University of Alabama at Birmingham, Birmingham, Alabama, USA

## Abstract

AChR is concentrated at the postjunctional membrane at the neuromuscular junction. However, the underlying mechanism is unclear. We show that α-actinin, a protein known to cross-link F-actin, interacts with rapsyn, a scaffold protein essential for neuromuscular junction formation. α-Actinin, rapsyn, and surface AChR form a ternary complex. Moreover, the rapsyn-α-actinin interaction is increased by agrin, a factor known to stimulate AChR clustering. Downregulation of α-actinin expression inhibits agrin-mediated AChR clustering. Furthermore, the rapsyn-α-actinin interaction can be disrupted by inhibiting Abl and by cholinergic stimulation. Together these results indicate a role for α-actinin in AChR clustering.

## Background

The concentration of nicotinic acetylcholine (ACh) receptors (AChRs) directly apposed to motoneuron terminals ensures the fast and efficient transmission from the nerve to the muscle. This concentration in the adult muscle fiber membrane results in an AChR density of 10,000 per micron at the postsynaptic membrane dropping to less than 10 per micron just a few micrometers away from the neuromuscular junction (NMJ) [1,2]. This is generated by complex interactions between motoneuron terminals and skeletal muscles [3-6]. Neural agrin clusters AChRs via activating the receptor complex consisting of LRP4 and MuSK [7-11], both of which are essential for NMJ formation [8,12]. ACh is thought to disassemble receptor clusters in non-synaptic areas via activating muscle fibers [13-15].

Rapsyn (for receptor-associated protein at synapse) is an intracellular peripheral protein that precisely co-localizes with the AChR [16-18]. Rapsyn is present at or greater than a 1:1 ratio with the AChR [19-21], and is believed to anchor AChRs at the synapse [18,21-26]. Recent studies suggest a multi-facet role of rapsyn in AChR clustering. It has been shown to interact with several proteins including the AChR [16-18,27], β-dystroglycan [28,29], actin [30], and β-catenin [31]. It is believed that these interactions bridge AChRs to the cytoskeleton. On the other hand, rapsyn prevents the activation of Cdk5, a kinase downstream of the negative signal ACh to disperse AChR clusters [32]. Using a proteomic approach to identify proteins that specifically associated with clustered surface AChR, we discovered a critical role of HSP90beta in AChR clustering by stabilizing rapsyn [33].

To further study rapsyn's role we performed a yeast two-hybrid screen with rapsyn as bait. One protein we found was α-actinin, an actin cross-linker. To study the possible role of α-actinin in AChR clustering, we carried out a number of experiments to characterize the interaction between α-actinin and rapsyn. We also investigated the consequences of suppressing α-actinin expression on AChR clustering. Finally, we looked at two factors that are known to disrupt agrin regulated AChR clustering to determine if they negatively regulate the rapsyn-α-actinin interaction. Results of these studies indicate a role for α-actinin in agrin-induced AChR clustering.

## Results

### Rapsyn and α-actinin interact and colocalize

**Figure 1:**
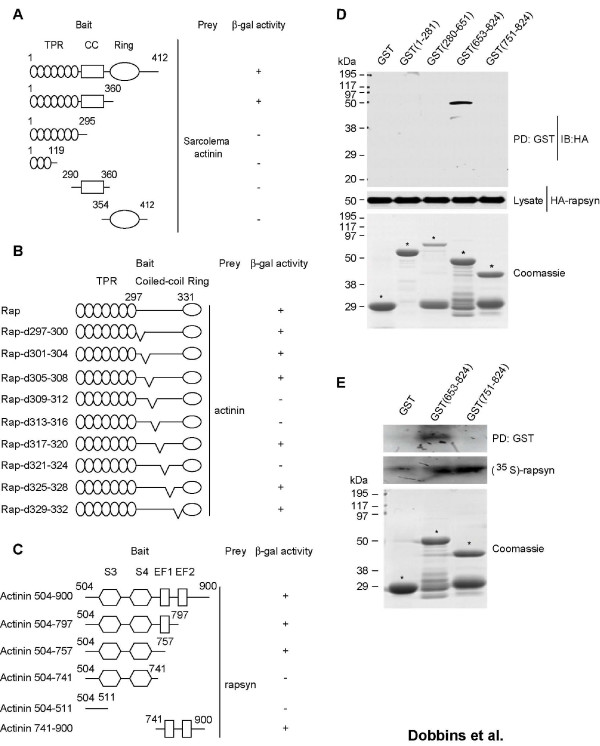
**α-Actinin interacts with rapsyn**. (A) Y190 cells were cotransformed with pGBT10-rapsyn and rapsyn mutants along with pACT2-α-actinin. Transformed yeast cells were seeded in Leu-Trp-His- plates and scored for β-gal activity: (-) no blue after 8 hr, (+) blue after 2 hr. The coiled-coil domain of rapsyn was required for its interaction with α-actinin. (B) Four amino acids at a time were mutated to cysteine starting at the beginning of rapsyn's coiled-coil domain. The mutants were then subcloned into pGBT10 and cotransformed into yeast cells with pACT2-α-actinin. Transformed yeast cells were seeded in Leu-Trp-His- plates and scored as in (A). Amino acids 309–316 and amino acids 321–324 within the coiled-coil domain were necessary for rapsyn's interaction with α-actinin. (C) pGBT10-raspyn was cotransformed with the pACT2-α-actinin constructs into yeast. A linker region composing amino acids 741–757 was required for the interaction with rapsyn. (D) α-Actinin GST-fusion proteins immobilized on glutathione sepharose 4B beads were incubated with lysates from HEK 293 cells transfected with HA-rapsyn. Bead-associated proteins were subjected to SDS-PAGE and immunoblotting (IB) with indicated antibodies. (E) [^35^S]-labeled rapsyn was generated by in vitro translation in the presence of [^35^S]-labeled methionine and incubated with bead-immobilized GST-α-actinin. Bound [^35^S]-labeled proteins were resolved on SDS-PAGE and subjected to autoradiogram. GST α-actinin (653–824) interacted directly with rapsyn.

To identify cytoskeletal proteins that may interact with rapsyn, we used the yeast two-hybrid system. A screen of a mouse cDNA library [34] using full length rapsyn as the bait found α-actinin. To determine which rapsyn domains were important for the interaction with α-actinin, we generated a number of rapysn mutants. Rapsyn has eight tetratricopeptide repeats (TPRs) (amino acids 6–319), a coiled-coil domain (amino acids 298–331), and a cysteine-rich domain (amino acids 363–402) [26,35]. The TPR domains are responsible for rapsyn self association; while the coiled-coil domain is required for AChR clustering and interacts with the AChR β-subunit cytoplasmic domain (reviewed by Banks et al., 2003)[36]. Hence, we generated rapsyn constructs lacking the ring domain, the coiled-coil domain and the TPR domain, and a construct that only included a few of the TPR domains. Constructs lacking the coiled-coil domain could not bind α-actinin, suggesting that this domain is required for interacting with α-actinin; however, the coiled-coil domain alone could not bind α-actinin, indicating that this region is not sufficient for the interaction (Fig. 1A).

Previous studies have shown that certain hydrophobic residues within rapsyn's coiled-coil domain were required for the protein's interaction with the AChR [27]. To explore which amino acids in the coiled-coil domain may be important for binding α-actinin, we generated a number of mutants in which four amino acids at a time were mutated to cysteine. Cotransformation of these mutants along with α-actinin into yeast cells showed that a number of regions within the coiled-coil domain were important in rapsyn's interaction with α-actinin. Specifically, amino acids 309–316 and amino acids 321–324 were required for the interaction (Fig. 1B). Hence, the coiled-coil domain is not only required for binding the AChR but is also important in rapsyn's interaction with α-actinin. Furthermore, the region that binds α-actinin included both hydrophobic and hydrophilic residues, indicating the region for α-actinin may be broader than that for AChR β-subunit [27].

**Figure 2:**
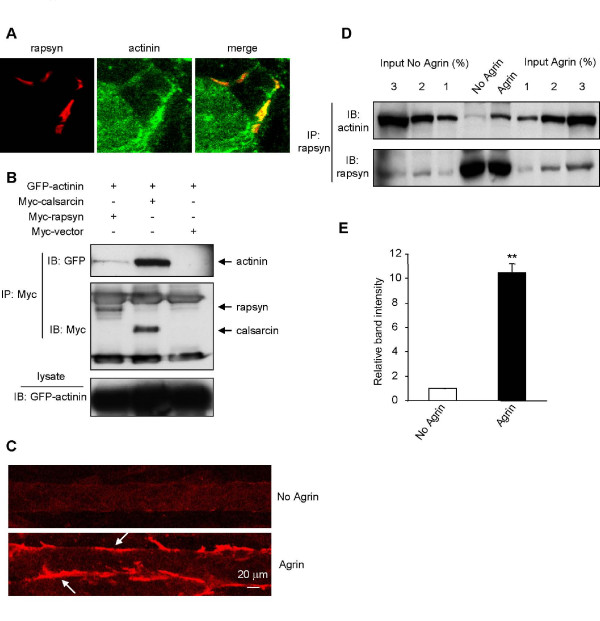
**Regulation of the rapsyn-α-actinin interaction by agrin**. (A) Colocalization of α-actinin and rapsyn. Cross sections of adult mouse diaphragm were costained with antibodies specific for α-actinin and rapsyn. α-Actinin and rapsyn immunoreactivity was visualized by FITC and Cy3 conjugated secondary antibodies, respectively. (B) HEK 293 cells were cotransfected with GFP-α-actinin, Myc-calsarcin, Myc-rapsyn or Myc empty vector. Cell lysates were incubated with anti-Myc antibody followed by protein G agarose beads. Resulting precipitates were probed by indicated antibodies. (C) Ability of agrin to stimulate AChR clustering. C2C12 myotubes were stimulated without (control) or with 10 ng/ml agrin for 18 hr, and stained with Alexa 594-conjugated α-BTX. (D) Increased interaction in agrin-stimulated C2C12 myotubes. Lysates of control and stimulated myotubes were incubated with a rapsyn specific antibody followed by protein A agarose beads. After separation by SDS-PAGE and membrane transfer, immunoblotting was carried out with an antibody specific to α-actinin. Percentage of lysate inputs was immunoblotted to quantify the immunoprecipitation. (E) Quantitation of blot. The amount of α-actinin interacting with rapsyn is significantly greater upon agrin treatment. Data represent mean ± SEM of three experiments. **, *p *< 0.01, two-tailed Student's paired *t *test.

Next, we identified which regions of α-actinin interact with rapsyn and whether or not the interaction was direct. α-Actinin forms anti-parallel dimers with an actin binding head, followed by four spectrin repeats leading to a calmodulin like domain comprising two EF hand repeats (reviewed by Otey and Carpen)[37]. Using a number of α-actinin mutants, we found that a linker region between the last spectrin domain and the first EF1 hand (amino acids 741–757) was required for the interaction with rapsyn in yeast cells (Fig. 1C). To confirm the finding, we generated a battery of α-actinin GST-fusion proteins and incubated the immobilized proteins with lysates of cells expressing HA-rapsyn. Bound rapsyn was probed with anti-HA antibodies. As shown in Fig. 1D, GST-fusion protein containing amino acids 653–824 interacted with HA-rapsyn, in agreement with observations from yeast studies. To determine whether the rapsyn-α-actinin interaction was direct, not via a third protein, α-actinin GST fusion proteins were incubated with [^35^S]-labeled rapsyn generated by in vitro translation. Again, [^35^S]-rapsyn bound to GST-α-actinin 653–824, but not the parental GST or α-actinin 751–824 (Fig. 1E). These results indicated that the two proteins directly bind to each other, and provide further evidence that the binding region in α-actinin is localized in a region between amino acids 653–751. Numerous studies have established that rapsyn precisely colocalizes with AChRs at the NMJ. Previous reports have also found that α-actinin and AChRs colocalize and associate at the NMJ [38,39]. Similarly, staining of mouse diaphragm sections with antibodies specific for α-actinin and rapsyn indicated colocalization between the two proteins (Fig. 2A).

### The rapsyn-α-actinin interaction is regulated by agrin

To determine whether rapsyn and α-actinin interact in mammalian cells, we cotransfected GFP-α-actinin with Myc-rapsyn, Myc-calsarcin (a known α-actinin binding partner [40]) or Myc-empty vector into HEK 293 cells. Immunoprecipitation of Myc-rapsyn resulted only in a weak coprecipitation of α-actinin, compared to calsarcin (Fig. 2B). Next we studied the rapsyn-α-actinin interaction by immunoprecipitating C2C12 myotubes with anti-rapsyn antibody and probing the precipitates for α-actinin. Similar to the results in HEK 293 cells, we only saw a weak association between the two proteins in untreated C2C12 cells. This led us to question whether the interaction may be regulated by agrin. Remarkably, when myotubes were stimulated with agrin for 18 hr that induce AChR clustering (Fig. 2C), we found a significant increase in coprecipitation between rapsyn and α-actinin (Fig. 2D and 2E). The interaction was increased about 6–10 fold in agrin-treated muscle cells, suggesting that agrin stimulates the rapsyn-α-actinin interaction.

### Rapsyn is required for the AChR-α-actinin interaction

**Figure 3:**
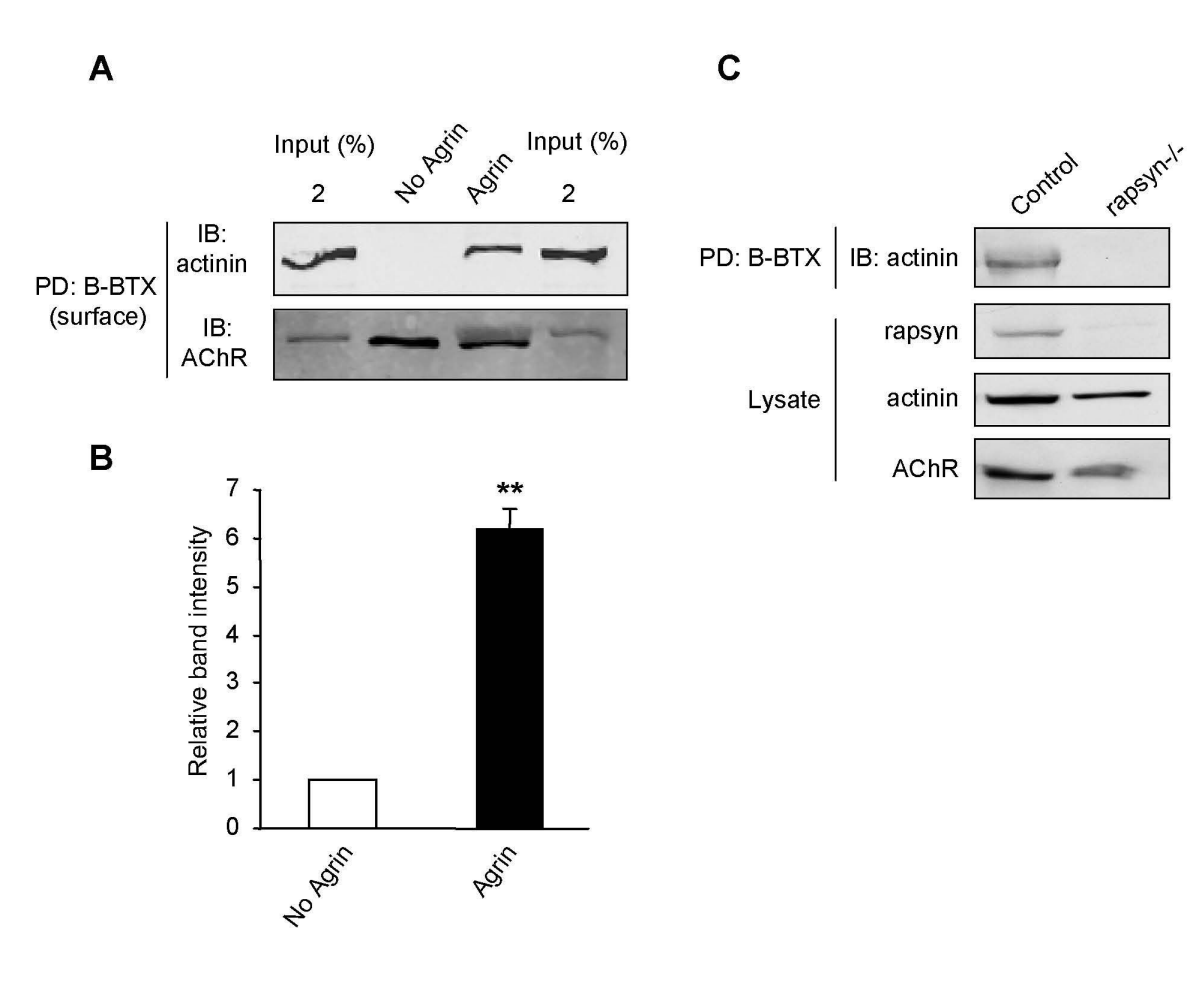
**A ternary complex of AChR, rapsyn, and α-actinin**. (A) Live C2C12 myotubes were incubated with B-BTX for 2 hr at 4°C. Surface receptors were purified by streptavidin-coupled agarose beads. AChR-associated proteins were probed with antibodies specific for α-actinin and the AChR β-subunit. (B) Quantitation of blot. The amount of α-actinin associated with surface AChRs was increased upon in agrin-stimulated myotubes. Data represent mean ± SEM of three experiments. **, *p *< 0.01, two-tailed Student's paired *t *test. (C) α-Actinin was not associated with AChRs in rapsyn -/- myotubes. Control and rapsyn mutant myotubes were treated with agrin. AChRs were labeled by B-BTX and probed for α-actinin.

The agrin-regulated rapsyn-α-actinin interaction does not necessarily imply that the AChRs also interact with α-actinin in an agrin-dependent manner. We used biotin-conjugated α-bungarotoxin (B-BTX) to pull down AChRs from C2C12 myotubes treated and untreated with agrin. Similar to the previous experiment, we found a significant increase in the AChR-α-actinin interaction following agrin stimulation (Fig. 3A and 3B).

The AChR-α-actinin interaction could be direct or mediated through another protein. To determine if the agrin regulated AChR-α-actinin interaction was dependent on rapsyn, control myotubes and rapsyn -/- myotubes were stimulated with agrin for 18 hr and AChRs were isolated as before. Interestingly, no α-actinin was detectable associated with AChRs in rapsyn -/- cells, unlike control myotubes (Fig. 3C), indicating that rapsyn is required for the AChR-α-actinin interaction. These results suggest that the α-actinin binding site in rapsyn may not overlap or interfere with that for AChRs and that α-actinin, rapsyn, and AChR may form a ternary complex upon agrin stimulation.

### A role for α-actinin in AChR clustering

**Figure 4:**
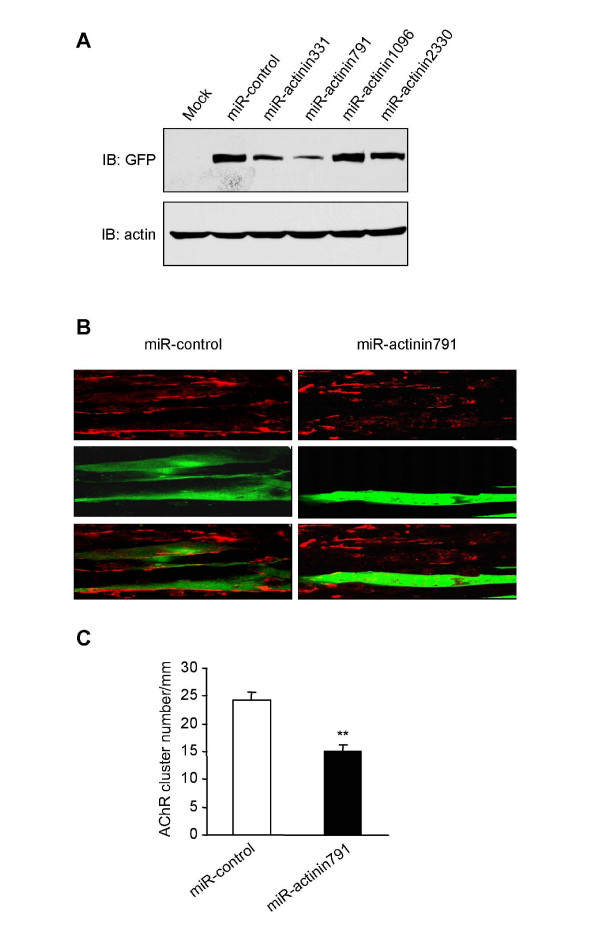
**Repression of α-actinin expression impaired agrin-induced AChR clustering**. (A) HEK293 cells were transfected with GFP-α-actinin along with indicated miR-RNAi constructs of α-actinin. Thirty-six hours after transfection, cell were lyzed and resulting lysates were immunoblotted with antibodies against GFP. miR-actinin791 showed the strongest inhibition of α-actinin expression. (B) Young C2C12 myotubes were transfected with the control vector or miR-RNA. After 24 hrs the myotubes were treated with agrin for 18 hr. AChR clusters were examined in myotubes expressing GFP that was encoded by the miR-RNAi parental vector. Agrin-induced AChR clusters were reduced in myotubes expressing miR-actinin791, but not miR-control encoding a scrambled sequence. Bar, 20 μm. (C) Quantitative analysis of data in (B). Data were presented as mean ± SEM, n = 20 each group; **, *P *< 0.01.

To investigate whether α-actinin is involved in AChR clustering, we used a micro-RNA (miRNA) approach to suppress its expression. Multiple double stranded oligo duplexes were designed targeting a number of regions toward α-actinin 2; miRNA-actinin791 in particular showed a substantial reduction of α-actinin 2 expression (Fig. 4A). We transfected this miRNA construct into myotubes and characterized agrin-induced AChR clusters. Transfected myotubes were labeled by GFP encoded by the parental miRNA construct. Compared to the control miRNA vector, we saw a significant reduction in AChR clusters in myotubes transfected with miRNA-actinin791 (Fig. 4B and 4C). Over-expression of α-actinin, however, had no influence on AChR clustering, probably because α-actinin is saturated *in vivo*.

**Figure 5:**
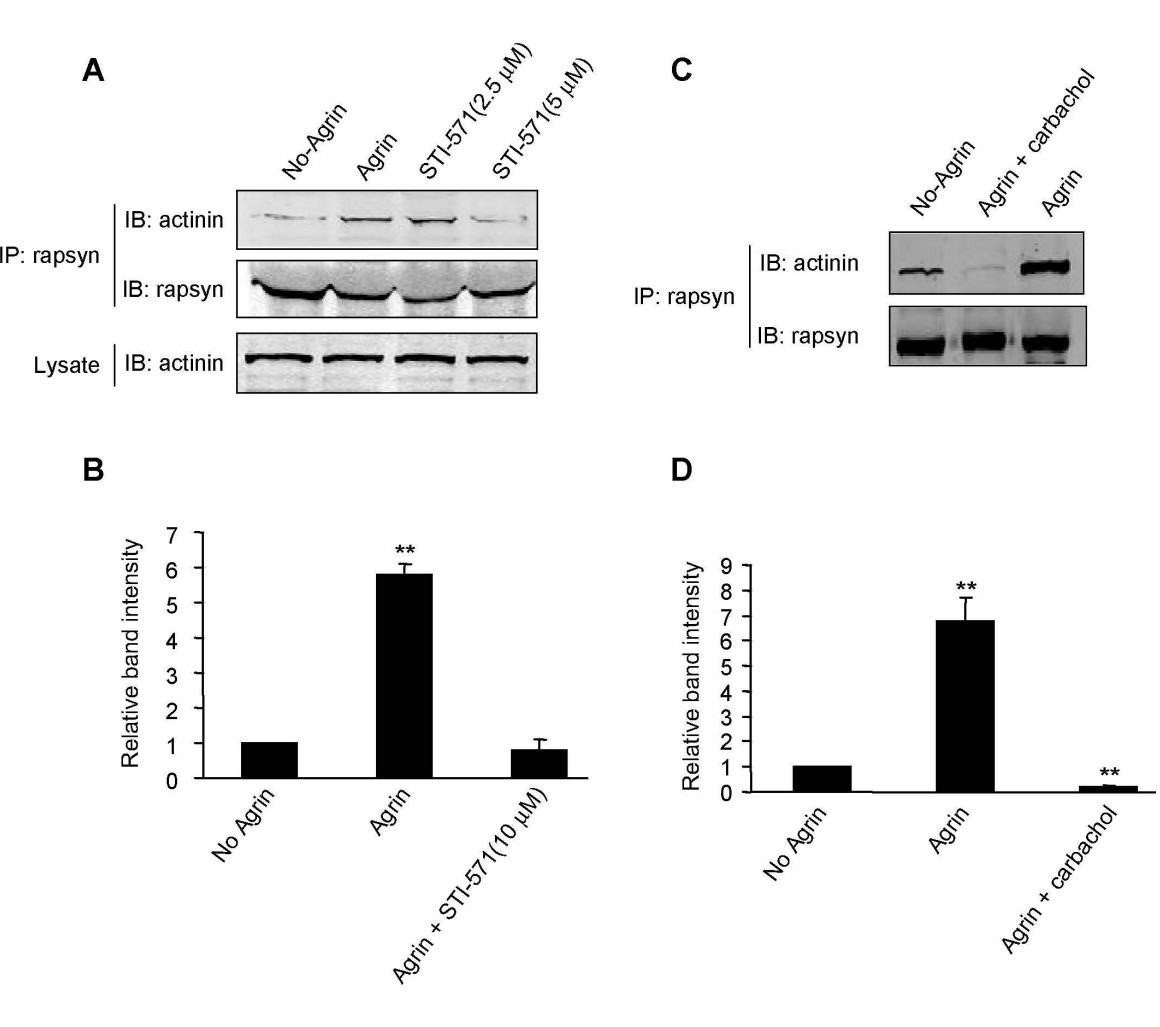
**Disruption of the rapsyn-α-actinin interaction by Abl inhibition and cholinergic stimulation**. (A) C2C12 myotubes were treated with agrin in the presence of different concentrations of STI-571. The interaction between rapsyn and α-actinin was characterized as in Fig. 2. (B) Quantification of (A). Data represent mean ± SEM of three experiments. **, *p *< 0.01, two-tailed Student's paired t test. (C) C2C12 myotubes were treated with or without agrin for 18 hr. After washing, agrin-treated myotubes were incubated with 10 μM carbachol. The carbachol treatment significantly decreased the rapsyn-α-actinin interaction not only compared to agrin-treated but also untreated myotubes. (D) Quantification of (C). Data represent mean ± SEM of three experiments. **, *p *< 0.01, by two-tailed Student's paired *t *test.

Abl represents a family of tyrosine kinases that among other functions regulate actin structure. They are localized at the NMJ, undergo reciprocal tyrosine phosphorylation with MuSK, and are required for agrin mediated AChR clustering [41]. We hypothesized that the rapsyn-α-actinin interaction is downstream of Abl and thus regulated by this tyrosine kinase. Hence, if the rapsyn-α-actinin interaction is important for AChR clustering, inhibiting Abl should disrupt the interaction. To test this hypothesis, myotubes were treated with agrin in the presence of STI-571, a specific inhibitor of Abl [41-43]. As previously reported [41], we found a significant reduction by STI-571 in the number of agrin-induced AChR clusters (data not shown). Intriguingly, the Abl inhibitor significantly reduced the rapsyn-α-actinin interaction (Fig. 5A and 5B).

Recent studies have shown that ACh and agrin play contrasting roles in shaping AChR clusters; agrin appears to offset ACh's dispersal mechanism [14,44,45]. To determine whether the rapsyn-α-actinin interaction is regulated by muscle activation, myotubes were first treated with agrin overnight, washed, and stimulated with carbachol, a non-hydrolysable cholinergic agonist, at 10 μM for 4 hr. As previously described, this treatment resulted in a dramatic reduction in the number of clusters (data not shown) [14,45,46]. Remarkably, there was a dramatic decrease in the rapsyn-α-actinin interaction in myotubes treated with carbachol.

## Discussion

In this study we find that α-actinin interacts with rapsyn; and the interaction was regulated by agrin. We also find that AChR, rapsyn, and α-actinin form a ternary complex that is also up-regulated by agrin. Furthermore, downregulating α-actinin expression inhibits agrin-mediated AChR clustering. Finally, we show that the rapsyn-α-actinin interaction can be disrupted by inhibiting Abl and by cholinergic stimulation. These results identify a role for α-actinin in agrin-induced AChR clustering.

α-Actinin is an important scaffolding protein known to cross-link F-actin. It forms head-to-tail dimmers via spectrin-like repeats creating a long molecule with actin binding domains at each end which cross-link actin filaments. In dendritic spines of hippocampal neurons, α-actinin has been found to interact directly with NMDA receptors, anchoring them to the underlying F-actin [47]. Also, α-actinin is thought to play a role in the formation and transport of AMPA receptors to the dendritic spines [48]. Our results indicate that the region responsible for α-actinin's interaction with rapsyn is within a linker region at the C terminus of the spectrin repeats. This finding is consistent with other studies that have mapped the importance of the spectrin domains and linker region in protein docking (reviewed by Otey and Carpen) [37]. α-Actinin has been shown to associate with AChRs in skeletal muscle [39]. We further characterize this association by isolating AChRs with B-BTX and probing for α-actinin. As with the rapsyn-α-actinin interaction, α-actinin is minimal in the AChR complex pulled down from untreated myotubes but is significantly increased in myotubes treated with agrin. The similarity between the agrin regulation of the rapsyn-α-actinin interaction and the AChR-α-actinin interaction strongly suggests that the AChR-α-actinin interaction is dependent on rapsyn. The fact that AChRs isolated from rapsyn -/- myotubes showed no interaction with actinin further supports the idea that rapsyn serves as a bridge between the AChRs and the cytoskeletal protein.

Abl has been implicated in AChR clustering, presumably downstream of MuSK. MuSK and Abl undergo reciprocal tyrosine phosphorylation upon agrin stimulation that is required for AChR clustering [41]. Abl is thought to be upstream of the Rho family of small GTPases [49,50]. Rac, Rho and Cdc 42 have all been shown to play a potentially important role in AChR clustering [51-53]. Results suggest that these proteins switch on actin polymerization, bringing actin to the site and or changing actin already at the site to aid AChR anchoring. We hypothesize that α-actinin is a regulatory scaffolding protein for the rapsyn-AChR complex, and may aid in this important cytoskeletal link. When Abl is inhibited, α-actinin losses its ability to interact with rapsyn in response to agrin stimulation. Consistently, activation of muscles by cholinergic agonist CCh disrupts the rapsyn-α-actinin interaction. Hence, the interaction is regulated by both positive, agrin, and negative, ACh, signals and may in part represent an important junction in AChR clustering.

How might the rapsyn-α-actinin interaction be controlled? Tyrosine kinases have been shown to regulate α-actinin's interaction with other proteins [54]. Agrin activation may lead to the phosphorylation of α-actinin, upregulating its interaction with rapsyn. This could work in tandem with actin modulation creating a scaffold for anchoring the rapsyn-AChR complex at the site of agrin deposition. Conversely, ACh may have the opposite effect. Studies suggest that ACh may mediate its dispersal effects through the serine-threonine kinase cdk5. Interestingly though, cdk5 is known to bind α-actinin [55]. An attractive hypothesis is that cdk5 is bound to α-actinin until agrin release results in a change of α-actinin binding partners from cdk5 to rapsyn. Alternatively, the rapsyn-α-actinin interaction could be regulated by a protein tyrosine phosphatase. There is evidence that AChR clustering and declustering are dependent on tyrosine kinases and phosphatses respectively [41,56-59]. Shp2 is concentrated at the NMJ [60] and has been implicated in AChR dispersal [56]. However, mutant mice that do not express Shp2 in muscle cells form apparently normal NMJs, suggesting that this phosphatase may be dispensable for NMJ formation and/or maintenance [61]. Interestingly, Shp1 has been found to be a phosphatase for α-actinin [62]. Whether Shp2 regulates AChR clustering or NMJ formation remains unclear because this phosphatase is enriched in heamopoitic cells.

Finally, α-actinin is enriched and concentrated in the postsynaptic density (PSD) of excitatory synapses in dendritic spines [47,63,64]. Here it binds NMDA and AMPA receptors and other PSD proteins linking them to the underlying actin cytoskeleton [48,65-67]. We have shown that the rapsyn-α-actinin interaction can be regulated by both positive and negative receptor clustering signals raising the possibility that α-actinin has a similar function in the PSD.

## Methods

### Reagents, constructs and antibodies

α-Actinin and mutant yeast constructs were subcloned into pACT2 or into pGEX-2T. Mouse rapsyn and mutant yeast constructs were subcloned into pGBT10. For mammalian cell expression, rapsyn was subcloned between Hind III and Xha I sites in pKH3, and between BamH I and EcoR I sites in pEF6/myc-his (Invitrogen). Rapsyn mutants were constructed with Quickchange Site-Directed Mutagenesis Kit (Stratagene, La Jolla, CA). Mammalian α-actinin plasmids were generous gifts from Dr. A. Woods (University of Alabama at Birmingham) and Dr. W. Hsu (National Defense Medical Center, Taiwan). The miRNAi entry vector was generated by the BLOCK-iT RNAi Expression System (Invitrogen) according to the manufacturer's instruction. The sequence for mouse α-actinin 2 was analyzed by a program provided by Invitrogen; four sequences were picked and cloned into pcDNA6.2-GW/EmGFP-miR to yield pcDNA6.2-GW/EmGFP-miR-α-actinin. The oligonucleotide sequences for miRNAi constructs were as follows: miRactinin331, 5'-TGC TGA ATA GAT ACC AGC TTC ACT CCG TTT TGG CCA CTG ACT GAC GGA GTG AAT GGT ATC TAT T (forward); 5'-CCT GAA TAG ATA CCA TTC ACT CCG TCA GTC AGT GGC CAA AAC GGA GTG AAG CTG GTA TCT ATT C (reverse). miRactinin791 5'-TGC TGT ACA TAT TCT GTT AGC TGC TGG TTT TGG CCA CTG ACT GAC CAG CAG CTC AGA ATA TGT A (forward); 5'-CCT GTA CAT ATT CTG AGC TGC TGG TCA GTC AGT GGC CAA AAC CAG CAG CTA ACA GAA TAT GTA C (reverse). miRactinin1096 5'-TGC TGA GCA ATG TCC GAC ACC ATC TTG TTT TGG CCA CTG ACT GAC AAG ATG GTC GGA CAT TGC T' (forward); 5'-CCT GAG CAA TGT CCG ACC ATC TTG TCA GTC AGT GGC CAA AAC AAG ATG GTG TCG GAC ATT GCT C (reverse). miRactinin2330 5'-TGC TGA AAT GAG GCA GGC TCT GAA ATG TTT TGG CCA CTG ACT GAC ATT TCA GAC TGC CTC ATT T (forward); 5'-CCT GAA ATG AGG CAG TCT GAA ATG TCA GTC AGT GGC CAA AAC ATT TCA GAG CCT GCC TCA TTT C (reverse). miRactinin2371 5'-TGC TGA ATG CGG GCA AAT TCA GCT TCG TTT TGG CCA CTG ACT GAC GAA GCT GAT TGC CCG CAT T (forward); 5'-CCT GAA TGC GGG CAA TCA GCT TCG TCA GTC AGT GGC CAA AAC GAA GCT GAA TTT GCC CGC ATT C (reverse). The numbers in the miRNA constructs indicate the start of targeted nucleotide sequences of the α-actinin 2 mouse gene.

Anti-α-actinin antibody was from Upstate (Charlottesville, VA); and fluorescein-conjugated cholera toxin B subunit (FITC-CTX) was from Sigma (St. Louis, MO). Rabbit anti-rapsyn antibody was generated using GST-fusion protein containing N-terminal fragment of mouse rapsyn. Myc and HA antibodies were from Santa Cruz. Anti-β-AChR antibody (mAb124) was a gift from Dr. J. Lindstrom (University of Pennsylvania Medical School, Philadelphia, PA). Interferon-gamma was from BioSource (Camarillo, CA). α-Bungarotoxin (α-BTX), biotin conjugated α-BTX (B-BTX), and Alexa Fluor 594-conjugated α-BTX were from Invitrogen (Eugene, OR). Neural agrin was prepared as described previously [31,52,68]. Agrin treatment was at 10 ng/ml for 18 hr which induced AChR clusters.

### Cell culture

C2C12 mouse muscle cells were maintained in a nutrient-rich growth medium containing DMEM supplemented with 2 mM L-glutamine, 20% fetal bovine serum, 0.5% chicken embryo extract, and 100 U/ml penicillin at 37°C in an atmosphere of 5% CO2 and 95% humidity [31,68]. Differentiation was induced when myoblasts were at 70% confluence by switching to the differentiation medium, DMEM supplemented with 4% horse serum, and 2 mM L-glutamine. Rapsyn -/- (clone 11–71) muscle cells were generously provided by Dr. Christian Fuhrer (University of Zurich, Zurich, Switzerland), and cultured as described previously [31,69]. Briefly, cells were maintained in the same basic medium as C2C12 cells, with an additional 4 U/ml interferon. These cells were then grown on dishes coated with 0.2% gelatin and maintained at 33°C with 5% CO2. To induce fusion, confluent cells were cultured in C2C12 fusion medium at 37°C, 5% CO2. In all experiments, the medium was replaced every day. HEK 293 cells were maintained as previously described [70]. Briefly, cells were maintained in a nutrient-rich growth medium containing DMEM supplemented with 2 mM L-glutamine, 10% fetal bovine serum, and 100 U/ml penicillin at 37°C in an atmosphere of 5% CO2 and 95% humidity. Transient transfection of cells was performed with the standard calcium phosphate technique or with lipofectamine 2000 according to the instructions of the manufacturer (Invitrogen, Carlsbad, CA).

### Immunoprecipitation, immunoblotting, and in vitro protein interactions

C2C12 myotubes or HEK 293 cells were rinsed twice with ice-cold PBS, solubilized for 15 min on ice in lysis buffer, containing 50 mM Tris-HCl (pH 7.5), 150 mM NaCl, 1% NP40, and .5% sodium deoxycholate supplemented with protease inhibitor coctail. Lysates were then centrifuged at 12,000 rpm for 10 min, and the supernatants were incubated with antibodies against rapsyn [68] or Myc at 4°C followed by protein A or protein G agarose beads respectively. To isolate surface AChRs, intact myotubes were incubated with biotin-conjugated α-BTX for 2 hr at 4°C. After washing, cells were lysed, and incubated with streptavidin-coupled agarose beads for 6 hr at 4°C [21,71]. After centrifugation, beads were washed twice with lysis buffer followed by two washes with a high salt lysis buffer and two times with a low salt lysis buffer. Bound proteins were eluted with SDS sample buffer and subjected to SDS-PAGE. Proteins resolved on SDS-PAGE were transferred to nitrocellulose Portran membranes (Schleicher and Schuell, Keene, NH), which were incubated at room temperature for 1 hr in blocking buffer (TBS with 0.1% Tween 20 and 5% milk), followed by an incubation with the necessary antibodies at 4°C overnight. After washing three times for 15 min each with TBS with 0.1% Tween 20, the blots were incubated with horseradish peroxidase-conjugated secondary antibody (Amersham Biosciences) or with Alexa Fluor 680-labeled anti-mouse IgG antibody (1:5000; Invitrogen, Eugene, OR), and IRDye 800-labeled anti-rabbit IgG antibody (1:3000; Rockland Immunochemicals, Gilbertsville, PA). Immunoreactive bands were then visualized using enhanced chemiluminescence substrate (Pierce, Rockford, IL) or the Odyssey imaging system (LI-COR, Lincoln, NE) following the manufacturer's protocols. In some experiments, after visualizing an immunoreactive protein, the nitrocellulose filter was incubated in a buffer containing 62.5 mM Tris/HCl, pH 6.7, 100 mM β-mercaptoethanol, and 2% SDS at 50°C for 30 min, washed with TBS with 0.1% Tween 20 at room temperature for 1 hr, and reblotted with different antibodies. For quantitative analysis, films were scanned with an Epson 1680 scanner, and the captured image was analyzed with NIH Image software or the Odyssey imaging system (LI-COR, Lincoln, NE) following the manufacturer's protocols.

α-Actinin 2 GST-fusion proteins were produced in BL21, purified, and immobilized on glutathione sepharose 4B beads (Amersham Pharmacia). They were then incubated with lysates from HEK293 cells transfected with HA-rapsyn. Bead-associated proteins were subjected to SDS-PAGE and immunoblotting analysis. To assay the direct interaction between rapsyn and α-actinin, [^35^S]-labeled rapsyn was generated by in vitro translation in the presence of [^35^S]-labeled methionine using TnT T7/SP6 Coupled Reticulocyte Lysate System (Promega, L5020) [53]. [^35^S]-labeled rapsyn was then incubated with immobilized GST-α-actinin constructs in the binding buffer (25 mM HEPES, 1 mM DTT, 0.5% Triton X-100,150 mM NaCl and protease inhibitors, pH7.5) for 2 hr at 4°C on a rotator. After washing with PBS/0.1% Tween 20, bound [^35^S]-labeled proteins were resolved on SDS-PAGE and subjected to autoradiogram.

### AChR clustering assays

Fully differentiated C2C12 myotubes, either control or treated, were incubated with soluble recombinant agrin to induce AChR clusters as described previously [31,68]. After fixation in 2% PFA for 30 min, cells were incubated with Alexa 594-conjugated α-BTX for 60 min to label AChR clusters. Myotube segments (200 μm in length) were viewed at 40× magnification with a Nikon (Tokyo, Japan) Optiphot microscope equipped with phase and epifluorescence optics; and the number of AChR aggregates was counted. AChR clusters with an axis >4 μm were counted in 10 fields of each dish. In some experiments, young myotubes (in differentiation medium for 2 days) were transfected with indicated constructs using the lipofectamine 2000 kit according to the manufacturer's instruction (Invitrogen, Carlsbad, CA). 24–36 hr later, transfected myotubes were subjected to AChR cluster assays and viewed under a Zeiss confocal laser scanning microscope (LSM 510 META 3.2).

## Abbreviations

AChR: nicotinic acetylcholine receptor; NMJ: neuromuscular junction; Rapsyn: receptor-associated protein at the synapse; Ach: acetylcholine; TPRs: tetratricopeptide; B-BTX: biotin conjugated α-bungarotoxin; MuSK: muscle-specific receptor tyrosine kinase.

## Authors' contributions

LM and WCX helped GCD design experiments. GCD, SWL YBL and ZHY performed experiments and statistical analysis. GCD and LM wrote the paper. All authors read and approved the final manuscript.

## References

[B1] Bevan S, Steinbach JH (1977). The distribution of alpha-bungarotoxin binding sites of mammalian skeletal muscle developing in vivo. J Physiol.

[B2] Merlie JP, Isenberg KE, Russell SD, Sanes JR (1984). Denervation supersensitivity in skeletal muscle: analysis with a cloned cDNA probe. J Cell Biol.

[B3] Fu AK, Cheung ZH, Ip NY (2008). Beta-catenin in reverse action. Nat Neurosci.

[B4] Li XM, Dong XP, Luo SW, Zhang B, Lee DH, Ting AK, Neiswender H, Kim CH, Carpenter-Hyland E, Gao TM (2008). Retrograde regulation of motoneuron differentiation by muscle beta-catenin. Nat Neurosci.

[B5] Sanes JR, Lichtman JW (2001). Induction, assembly, maturation and maintenance of a postsynaptic apparatus. Nat Rev Neurosci.

[B6] Schaeffer L, de Kerchove d'Exaerde A, Changeux JP (2001). Targeting transcription to the neuromuscular synapse. Neuron.

[B7] Zhang B, Luo S, Wang Q, Suzuki T, Xiong WC, Mei L (2008). LRP4 serves as a coreceptor of agrin. Neuron.

[B8] DeChiara TM, Bowen DC, Valenzuela DM, Simmons MV, Poueymirou WT, Thomas S, Kinetz E, Compton DL, Rojas E, Park JS (1996). The receptor tyrosine kinase MuSK is required for neuromuscular junction formation in vivo. Cell.

[B9] Gautam M, Noakes PG, Moscoso L, Rupp F, Scheller RH, Merlie JP, Sanes JR (1996). Defective neuromuscular synaptogenesis in agrin-deficient mutant mice. Cell.

[B10] Kim N, Stiegler AL, Cameron TO, Hallock PT, Gomez AM, Huang JH, Hubbard SR, Dustin ML, Burden SJ (2008). Lrp4 Is a Receptor for Agrin and Forms a Complex with MuSK. Cell.

[B11] McMahan UJ, Horton SE, Werle MJ, Honig LS, Kroger S, Ruegg MA, Escher G (1992). Agrin isoforms and their role in synaptogenesis. Curr Opin Cell Biol.

[B12] Weatherbee SD, Anderson KV, Niswander LA (2006). LDL-receptor-related protein 4 is crucial for formation of the neuromuscular junction. Development.

[B13] Brandon EP, Lin W, D'Amour KA, Pizzo DP, Dominguez B, Sugiura Y, Thode S, Ko CP, Thal LJ, Gage FH (2003). Aberrant patterning of neuromuscular synapses in choline acetyltransferase-deficient mice. J Neurosci.

[B14] Lin W, Dominguez B, Yang J, Aryal P, Brandon EP, Gage FH, Lee KF (2005). Neurotransmitter acetylcholine negatively regulates neuromuscular synapse formation by a Cdk5-dependent mechanism. Neuron.

[B15] Misgeld T, Burgess RW, Lewis RM, Cunningham JM, Lichtman JW, Sanes JR (2002). Roles of neurotransmitter in synapse formation: development of neuromuscular junctions lacking choline acetyltransferase. Neuron.

[B16] Burden SJ, DePalma RL, Gottesman GS (1983). Crosslinking of proteins in acetylcholine receptor-rich membranes: association between the beta-subunit and the 43 kd subsynaptic protein. Cell.

[B17] Sealock R, Wray BE, Froehner SC (1984). Ultrastructural localization of the Mr 43,000 protein and the acetylcholine receptor in Torpedo postsynaptic membranes using monoclonal antibodies. J Cell Biol.

[B18] Noakes PG, Phillips WD, Hanley TA, Sanes JR, Merlie JP (1993). 43 K protein and acetylcholine receptors colocalize during the initial stages of neuromuscular synapse formation in vivo. Dev Biol.

[B19] Frail DE, Mudd J, Shah V, Carr C, Cohen JB, Merlie JP (1987). cDNAs for the postsynaptic 43-kDa protein of Torpedo electric organ encode two proteins with different carboxyl termini. Proc Natl Acad Sci USA.

[B20] LaRochelle WJ, Froehner SC (1986). Determination of the tissue distributions and relative concentrations of the postsynaptic 43-kDa protein and the acetylcholine receptor in Torpedo. J Biol Chem.

[B21] Moransard M, Borges LS, Willmann R, Marangi PA, Brenner HR, Ferns MJ, Fuhrer C (2003). Agrin regulates rapsyn interaction with surface acetylcholine receptors, and this underlies cytoskeletal anchoring and clustering. J Biol Chem.

[B22] Apel ED, Merlie JP (1995). Assembly of the postsynaptic apparatus. Curr Opin Neurobiol.

[B23] Bloch RJ, Froehner SC (1987). The relationship of the postsynaptic 43 K protein to acetylcholine receptors in receptor clusters isolated from cultured rat myotubes. J Cell Biol.

[B24] Gautam M, Noakes PG, Mudd J, Nichol M, Chu GC, Sanes JR, Merlie JP (1995). Failure of postsynaptic specialization to develop at neuromuscular junctions of rapsyn-deficient mice. Nature.

[B25] Maimone MM, Merlie JP (1993). Interaction of the 43 kd postsynaptic protein with all subunits of the muscle nicotinic acetylcholine receptor. Neuron.

[B26] Ramarao MK, Cohen JB (1998). Mechanism of nicotinic acetylcholine receptor cluster formation by rapsyn. Proc Natl Acad Sci USA.

[B27] Ramarao MK, Bianchetta MJ, Lanken J, Cohen JB (2001). Role of rapsyn tetratricopeptide repeat and coiled-coil domains in self-association and nicotinic acetylcholine receptor clustering. J Biol Chem.

[B28] Cartaud A, Coutant S, Petrucci TC, Cartaud J (1998). Evidence for in situ and in vitro association between beta-dystroglycan and the subsynaptic 43 K rapsyn protein. Consequence for acetylcholine receptor clustering at the synapse. J Biol Chem.

[B29] Bartoli M, Ramarao MK, Cohen JB (2001). Interactions of the rapsyn RING-H2 domain with dystroglycan. J Biol Chem.

[B30] Antolik C, Catino DH, O'Neill AM, Resneck WG, Ursitti JA, Bloch RJ (2007). The actin binding domain of ACF7 binds directly to the tetratricopeptide repeat domains of rapsyn. Neuroscience.

[B31] Zhang B, Luo S, Dong XP, Zhang X, Liu C, Luo Z, Xiong WC, Mei L (2007). Beta-catenin regulates acetylcholine receptor clustering in muscle cells through interaction with rapsyn. J Neurosci.

[B32] Chen F, Qian L, Yang ZH, Huang Y, Ngo ST, Ruan NJ, Wang J, Schneider C, Noakes PG, Ding YQ (2007). Rapsyn interaction with calpain stabilizes AChR clusters at the neuromuscular junction. Neuron.

[B33] Luo S, Zhang B, Dong XP, Tao Y, Ting A, Zhou Z, Meixiong J, Luo J, Chiu FC, Xiong WC (2008). HSP90beta regulates rapsyn turnover and subsequent AChR cluster formation and maintenance. Neuron.

[B34] Lumeng C, Phelps S, Crawford GE, Walden PD, Barald K, Chamberlain JS (1999). Interactions between beta 2-syntrophin and a family of microtubule-associated serine/threonine kinases. Nat Neurosci.

[B35] Ponting CC, Phillips C (1996). Rapsyn's knobs and holes: eight tetratrico peptide repeats. Biochem J.

[B36] Banks GB, Fuhrer C, Adams ME, Froehner SC (2003). The postsynaptic submembrane machinery at the neuromuscular junction: requirement for rapsyn and the utrophin/dystrophin-associated complex. J Neurocytol.

[B37] Otey CA, Carpen O (2004). Alpha-actinin revisited: a fresh look at an old player. Cell Motil Cytoskeleton.

[B38] Bloch RJ, Hall ZW (1983). Cytoskeletal components of the vertebrate neuromuscular junction: vinculin, alpha-actinin, and filamin. J Cell Biol.

[B39] Mitsui T, Kawajiri M, Kunishige M, Endo T, Akaike M, Aki K, Matsumoto T (2000). Functional association between nicotinic acetylcholine receptor and sarcomeric proteins via actin and desmin filaments. J Cell Biochem.

[B40] Frey N, Richardson JA, Olson EN (2000). Calsarcins, a novel family of sarcomeric calcineurin-binding proteins. Proc Natl Acad Sci USA.

[B41] Finn AJ, Feng G, Pendergast AM (2003). Postsynaptic requirement for Abl kinases in assembly of the neuromuscular junction. Nat Neurosci.

[B42] O'Dwyer ME, Mauro MJ, Druker BJ (2002). Recent advancements in the treatment of chronic myelogenous leukemia. Annu Rev Med.

[B43] Okuda K, Weisberg E, Gilliland DG, Griffin JD (2001). ARG tyrosine kinase activity is inhibited by STI571. Blood.

[B44] Fu AK, Ip FC, Fu WY, Cheung J, Wang JH, Yung WH, Ip NY (2005). Aberrant motor axon projection, acetylcholine receptor clustering, and neurotransmission in cyclin-dependent kinase 5 null mice. Proc Natl Acad Sci USA.

[B45] Misgeld T, Kummer TT, Lichtman JW, Sanes JR (2005). Agrin promotes synaptic differentiation by counteracting an inhibitory effect of neurotransmitter. Proc Natl Acad Sci USA.

[B46] Bloch RJ (1986). Loss of acetylcholine receptor clusters induced by treatment of cultured rat myotubes with carbachol. J Neurosci.

[B47] Nakagawa T, Engler JA, Sheng M (2004). The dynamic turnover and functional roles of alpha-actinin in dendritic spines. Neuropharmacology.

[B48] Schulz TW, Nakagawa T, Licznerski P, Pawlak V, Kolleker A, Rozov A, Kim J, Dittgen T, Kohr G, Sheng M (2004). Actin/alpha-actinin-dependent transport of AMPA receptors in dendritic spines: role of the PDZ-LIM protein RIL. J Neurosci.

[B49] Lanier LM, Gertler FB (2000). From Abl to actin: Abl tyrosine kinase and associated proteins in growth cone motility. Curr Opin Neurobiol.

[B50] Liebl EC, Forsthoefel DJ, Franco LS, Sample SH, Hess JE, Cowger JA, Chandler MP, Shupert AM, Seeger MA (2000). Dosage-sensitive, reciprocal genetic interactions between the Abl tyrosine kinase and the putative GEF trio reveal trio's role in axon pathfinding. Neuron.

[B51] Weston CA, Teressa G, Weeks BS, Prives J (2007). Agrin and laminin induce acetylcholine receptor clustering by convergent, Rho GTPase-dependent signaling pathways. J Cell Sci.

[B52] Luo Z, Wang Q, Dobbins GC, Levy S, Xiong WC, Mei L (2003). Signaling complexes for postsynaptic differentiation. J Neurocytol.

[B53] Luo ZG, Wang Q, Zhou JZ, Wang J, Luo Z, Liu M, He X, Wynshaw-Boris A, Xiong WC, Lu B (2002). Regulation of AChR clustering by Dishevelled interacting with MuSK and PAK1. Neuron.

[B54] MacArthur DG, North KN (2004). A gene for speed? The evolution and function of alpha-actinin-3. Bioessays.

[B55] Dhavan R, Greer PL, Morabito MA, Orlando LR, Tsai LH (2002). The cyclin-dependent kinase 5 activators p35 and p39 interact with the alpha-subunit of Ca2+/calmodulin-dependent protein kinase II and alpha-actinin-1 in a calcium-dependent manner. J Neurosci.

[B56] Madhavan R, Zhao XT, Ruegg MA, Peng HB (2005). Tyrosine phosphatase regulation of MuSK-dependent acetylcholine receptor clustering. Mol Cell Neurosci.

[B57] Mohamed AS, Rivas-Plata KA, Kraas JR, Saleh SM, Swope SL (2001). Src-class kinases act within the agrin/MuSK pathway to regulate acetylcholine receptor phosphorylation, cytoskeletal anchoring, and clustering. J Neurosci.

[B58] Mittaud P, Camilleri AA, Willmann R, Erb-Vogtli S, Burden SJ, Fuhrer C (2004). A single pulse of agrin triggers a pathway that acts to cluster acetylcholine receptors. Mol Cell Biol.

[B59] Dai Z, Peng HB (1998). A role of tyrosine phosphatase in acetylcholine receptor cluster dispersal and formation. J Cell Biol.

[B60] Tanowitz M, Si J, Yu DH, Feng GS, Mei L (1999). Regulation of neuregulin-mediated acetylcholine receptor synthesis by protein tyrosine phosphatase SHP2. J Neurosci.

[B61] Dong XP, Li XM, Gao TM, Zhang EE, Feng GS, Xiong WC, Mei L (2006). Shp2 is dispensable in the formation and maintenance of the neuromuscular junction. Neurosignals.

[B62] Lin SY, Raval S, Zhang Z, Deverill M, Siminovitch KA, Branch DR, Haimovich B (2004). The protein-tyrosine phosphatase SHP-1 regulates the phosphorylation of alpha-actinin. J Biol Chem.

[B63] Walikonis RS, Jensen ON, Mann M, Provance DW, Mercer JA, Kennedy MB (2000). Identification of proteins in the postsynaptic density fraction by mass spectrometry. J Neurosci.

[B64] Peng J, Kim MJ, Cheng D, Duong DM, Gygi SP, Sheng M (2004). Semiquantitative proteomic analysis of rat forebrain postsynaptic density fractions by mass spectrometry. J Biol Chem.

[B65] Wyszynski M, Lin J, Rao A, Nigh E, Beggs AH, Craig AM, Sheng M (1997). Competitive binding of alpha-actinin and calmodulin to the NMDA receptor. Nature.

[B66] Dunah AW, Wyszynski M, Martin DM, Sheng M, Standaert DG (2000). alpha-actinin-2 in rat striatum: localization and interaction with NMDA glutamate receptor subunits. Brain Res Mol Brain Res.

[B67] Nuriya M, Oh S, Huganir RL (2005). Phosphorylation-dependent interactions of alpha-Actinin-1/IQGAP1 with the AMPA receptor subunit GluR4. J Neurochem.

[B68] Zhu D, Xiong WC, Mei L (2006). Lipid rafts serve as a signaling platform for nicotinic acetylcholine receptor clustering. J Neurosci.

[B69] Fuhrer C, Gautam M, Sugiyama JE, Hall ZW (1999). Roles of rapsyn and agrin in interaction of postsynaptic proteins with acetylcholine receptors. J Neurosci.

[B70] Huang YZ, Won S, Ali DW, Wang Q, Tanowitz M, Du QS, Pelkey KA, Yang DJ, Xiong WC, Salter MW (2000). Regulation of neuregulin signaling by PSD-95 interacting with ErbB4 at CNS synapses. Neuron.

[B71] Yang XL, Huang YZ, Xiong WC, Mei L (2005). Neuregulin-induced expression of the acetylcholine receptor requires endocytosis of ErbB receptors. Mol Cell Neurosci.

